# Tangential Flow Filtration for Highly Efficient Concentration of Extracellular Vesicles from Large Volumes of Fluid

**DOI:** 10.3390/cells7120273

**Published:** 2018-12-16

**Authors:** Sara Busatto, George Vilanilam, Taylor Ticer, Wen-Lang Lin, Dennis W. Dickson, Shane Shapiro, Paolo Bergese, Joy Wolfram

**Affiliations:** 1 Department of Transplantation Medicine; Department of Physiology and Biomedical Engineering, Mayo Clinic, Jacksonville, FL 32224, USA; vilanilam.george@mayo.edu (G.V.); taylorticer33@gmail.com (T.T.); 2 Department of Molecular and Translational Medicine, University of Brescia, 25123 Brescia, Italy; 3 Department of Neuroscience, Mayo Clinic, Jacksonville, FL 32224, USA; lin.wenlang@mayo.edu (W.-L.L.); dickson.dennis@mayo.edu (D.W.D.); 4 Department of Orthopedic Surgery, Mayo Clinic, Jacksonville, FL 32224, USA; shapiro.shane@mayo.edu; 5 CSGI, Research Center for Colloids and Nanoscience, 50019 Florence, Italy; 6 Department of Nanomedicine, Houston Methodist Research Institute, Houston, TX 77030, USA

**Keywords:** exosome, extracellular vesicles, isolation, ultrafiltration, tangential flow filtration, lipoaspirate

## Abstract

Concentration of extracellular vesicles (EVs) from biological fluids in a scalable and reproducible manner represents a major challenge. This study reports the use of tangential flow filtration (TFF) for the highly efficient isolation of EVs from large volumes of samples. When compared to ultracentrifugation (UC), which is the most widely used method to concentrate EVs, TFF is a more efficient, scalable, and gentler method. Comparative assessment of TFF and UC of conditioned cell culture media revealed that the former concentrates EVs of comparable physicochemical characteristics, but with higher yield, less single macromolecules and aggregates (<15 nm in size), and improved batch-to-batch consistency in half the processing time (1 h). The TFF protocol was then successfully implemented on fluids derived from patient lipoaspirate. EVs from adipose tissue are of high clinical relevance, as they are expected to mirror the regenerative properties of the parent cells.

## 1. Introduction

Extracellular vesicles (EVs) are cell-secreted nanoparticles that are primarily involved in inter-cellular communication processes [1]. In the past few years, EVs have garnered increasing interest from the scientific community, becoming a multidisciplinary field of research. EVs are characterized by unique physicochemical properties that arise due to nanosized dimensions and bioactive content. EVs are able to cross several biological barriers, such as the cell membrane and blood-brain barrier, and they are involved in many pathological processes [2]. For these reasons, EVs have promising applications as drug targets [3], therapeutic agents [4], biomarkers [5,6], and delivery vehicles [7,8,9].

A major challenge for the use of EVs for scientific and medical applications is the isolation process, as EVs can be found in various complex biological matrices that are composed of a plethora of nanosized biomaterials that overlap in size and density [10]. Common isolation techniques for the separation of EVs from other fluid components are ultracentrifugation (UC) [11,12], density gradients (DG) [13], and size exclusion chromatography (SEC) [14]. However, these methods can be limited by input volume, time consumption, and EV yield. For example, UC protocols are able to process relatively large volumes (up to 1.5 L), but they result in low recovery rates [15,16], have time-consuming centrifugation steps [17,18], frequently damage the EV structure [18,19], and cause the coprecipitation of contaminants [20]. Although DG and SEC usually result in EV formulations with high purity, these protocols are time-consuming, result in poor yields, and require small volumes (less than 5 mL) [13,17,18]. Moreover, clinical translation of the above-mentioned methods is challenging due to sterility and scale-up requirements for clinical-grade manufacturing. Therefore, improved techniques that enable EV concentration in a scalable, reproducible, and sterile manner, are necessary for future clinical translation.

**Figure 1:**
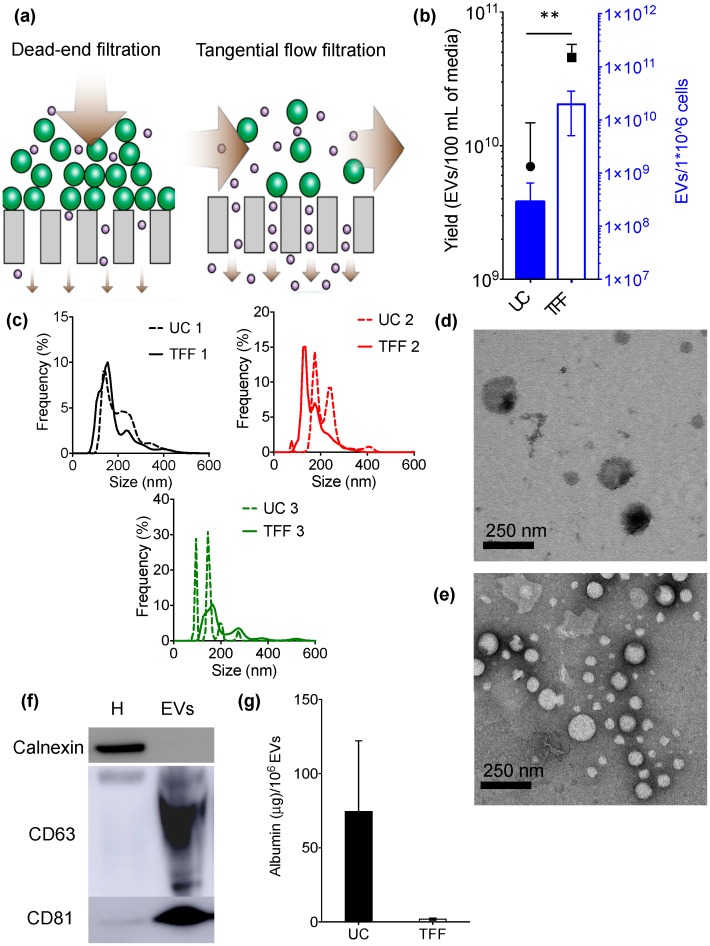
Biophysical and biochemical characterization of extracellular vesicles (EVs) isolated from MDA-MB-231 breast cancer cell culture media using ultracentrifugation (UC) or tangential flow filtration (TFF). (**a**) Schematic illustrating the difference between dead-end filtration and TFF; (**b**) EV yield; (**c**) Size distribution (0–650 nm) of EVs isolated with UC (dashed lines) and TFF (continuous lines); (**d**,**e**) Transmission electron microscopy (TEM) of EVs obtained with UC (**d**) or TFF (**e**); (**f**) Western blot of characteristic intracellular (calnexin) and EV (CD63, CD81) markers in samples isolated by TFF; (**g**) Levels of albumin contaminants in EV samples. Cellular homogenate (H) was used as a control. Data are presented as mean ± s.d. of three biological replicates (**b**,**c**) or experimental replicates (**g**). Statistical significance was evaluated by Student’s *t*-test. ** *p* < 0.01.

In the past years, techniques that are utilized for purification of synthetic nanoparticles, proteins, and viruses from liquid suspensions have also been adapted for EV separation. These emerging EV isolation methods primarily exploit antigen-antibody binding affinity, high-performance liquid chromatography, or filtration. For example, field flow fractionation (FFF) [21,22] and asymmetrical flow field-flow fractionation (AF4) [23,24] rely on a field applied to a liquid suspension that flows through tubular filters. Tangential flow filtration is another emerging technique that couples permeable membrane filtration and flow to obtain an efficient concentration of EVs from a colloidal matrix. Tangential flow filtration (TFF) differs from conventional dead-end filtration, as fluid flows tangentially across the surface, avoiding filter cake formation (Figure 1a). In fact, with dead-end filtration, larger particles usually clog the pores of the membrane, leading to impaired particle separation that is based on the original pore size.

In this study, TFF was evaluated as a size-based EV concentration method that is capable of processing scalable volumes of biological fluids. A side-by-side comparison of UC and TFF for processing large volumes of conditioned cell culture media was performed. Finally, the ability of TFF to process fluids derived from patient lipoaspirate was assessed. Among various clinically relevant biological matrices, adipose tissue is of particular relevance. Adipose tissue regulates several metabolic processes and is a source of mesenchymal stem cells that secrete a broad selection of soluble factors and EVs with regenerative properties [25,26].

## 2. Materials and Methods

### 2.1. Cell Culture

MDA-MB-231 human breast cancer cells (ATCC, HTB-26) and human primary brain microvascular endothelial cells (Cell System, ACBRI 376) were cultured at 37 °C in 5% CO_2_ and used at passage 2–20. MDA-MB-231 cells were maintained in high glucose Dulbecco’s modified eagle’s medium (DMEM) (Life Technologies, Gaithersburg, MD, USA), supplemented with 10% fetal bovine serum (FBS) (Sigma, St. Louis, MO, USA), 1% penicillin/streptomycin (Gemini Bioproducts, West Sacramento, CA, USA), and 1% glutamine (Life Technologies), while endothelial cells were maintained in Complete Classic Medium Kit with Serum and Culture Boost (Cell Systems, USA). For EV isolation, MDA-MB-231 cells were seeded in 150 mm dishes with DMEM supplemented with 10% exosome-depleted FBS (System Biosciences, Mountain View, CA, USA). The media was collected after 48 h when the cells were 90% confluent with a viability of >95% (Trypan blue). The media was centrifuged (800× *g*; 30 min; Sorvall ST 16R centrifuge, Thermo Fisher Scientific, San Jose, CA, USA) to discard the dead cells and large cellular debris.

### 2.2. Ultracentrifugation Protocol

Cell culture media (200 mL) underwent ultracentrifugation, as previously described (100,000× *g*; 70 min; Optima L100XP ultracentrifuge, Type Ti 70 rotor k factor 44, Beckman Coulter, San Diego, CA, USA) [27]. The pellet was then suspended in phosphate buffered saline (PBS; 2 mL) and the ultracentrifugation step was repeated to obtain an EV pellet that was suspended in 200 µL of sucrose buffer (5% sucrose, 50 mM Tris, and 2 mM MgCl) and analyzed.

### 2.3. Lipoaspirate Samples

Following institutional review board approval, de-identified lipoaspirate waste samples from patients that had underwent liposuction were processed with the non-enzymatic micro-fragmented process Lipogems (Atlanta, GA, USA) that mechanically disrupts fresh adipose tissue harvested via lipoaspiration [28]. The micro-fragmentation occurs via mechanical disruption using metal bearings and sterile saline. As the mechanical process pushes the micro-fragmented adipose tissue through a series of successive size reducing clusters, the elimination of waste products is captured in a sterile waste bag that is connected to the micro-fragmentation device cylinder. The waste bag content from the Lipogems device was transferred to tubes and centrifuged (800× *g*; 30 min; Sotvall ST 16R centrifuge, Thermo Scientific) to remove large debris. The supernatant was collected, diluted 1:1 in sucrose buffer (5% sucrose, 50 mM Tris, and 2 mM MgCl) in order to facilitate TFF filtration (avoid clogging), and immediately processed according to the TFF protocol reported below in order to concentrate EVs. Sucrose buffer is a commonly used suspension buffer and cryoprotectant for oncolytic measles viruses in clinical trials [29,30,31,32]. Measles viruses are similar in size, shear sensitivity, and lipid membrane characteristics as mammalian EVs [33], suggesting that this buffer could be suitable for clinical-grade EV preparations.

### 2.4. TFF

EVs were isolated using a KrosFlo Research 2i Tangential Flow Filtration System (Spectrum Labs., Los Angeles, CA, USA). Cell culture media (0.2 L) or lipoaspirate (1 L) were filtered using sterile hollow fiber polyethersulfone membranes with 0.65 μm (D02-E65U-07-S; Spectrum Labs) and 500 kD (D02-S500-05-S; Spectrum Labs) molecular weight cut-off pores to remove cell debris and free biomolecules, respectively. Filters were first washed with three times the volume of sterile PBS (pH 7.4) and the biological samples were then processed. The input flow rate was 80 mL/min in order keep the shear force of the feed stream below 2000 s^−1^. EVs were concentrated to approximately 50 mL and diafiltrated six times in sucrose buffer (5% sucrose, 50 mM Tris, and 2 mM MgCl). The final EV-solution in sucrose buffer was concentrated to reach a final volume of 6–9 mL and then analyzed.

### 2.5. Nanoparticle Tracking Analysis

The size and concentration of isolated EV samples were determined with nanoparticle tracking analysis. EV formulations were diluted (1:100–1:1000) in sterile phosphate buffered saline (PBS) and analyzed (500 µL) with a Nanosight NS300 (60 s measurement; three capture replicates, Malvern Panalytical, Westborough, MA, USA).

### 2.6. Transmission Electron Microscopy

EVs were fixed in 4% paraformaldehyde (1:1) at room temperature (2 min), placed (2 μL) on carbon-formvar-coated copper grids (300 mesh), and blotted. Water (2 μL) was added to the samples that were then blotted, and 2% aqueous uranyl acetate (2 μL) was placed on the grid (2 min) and blotted. The grids were examined with a 208S Electron Microscope (FEI; 60 kV, Philips, Amsterdam, NY, USA). Digital images were obtained with an 831 Orius Camera (Gatan, Pleasanton, CA, USA) and were processed with Adobe Photoshop CS5 (64 bit) software.

### 2.7. Western Blot

For the identification of common EV markers, samples that were isolated by TFF were mixed with reducing sample buffer (480 mM Tris at pH 6.8, 12% sodium dodecyl sulfate, 45% glycerine, 0.06% bromophenol blue, and 12% 2-mercaptoethanol) and boiled (5 min; 95 °C). MDA-MB-231 (cell culture media study) and brain endothelial cell (lipoaspirate study) homogenates were used as controls. Homogenates were obtained through tip sonication (5 s; 20% potency), followed by centrifugation (800× *g*; 10 min; 5417R Refrigerated Centrifuge, Eppendorf, Westbury, NY, USA). The protein content of the samples was measured with a BCA protein assay kit (Pierce, Thermo Fisher Scientific), and the samples (20–50 μg protein) were electrophoresed on a polyacrylamide gel (4–12%; Invitrogen) and analyzed by Western Blot. The following antibodies were used: rabbit monoclonal anti-CD63 (1:1000 dilution; clone ab134045; Abcam, Cambridge, MA, USA), mouse monoclonal anti-annexin V (1:500 dilution; clone ab54775; Abcam), mouse anti-CD9 (1:500 dilution; clone ab2215; Abcam), mouse monoclonal anti-CD81 (1:500 dilution; clone (B11): sc-166029; Santa Cruz, Santa Cruz, CA, USA), rabbit polyclonal anti-calnexin (1:1000 dilution; clone ab10286; Abcam), anti-rabbit IgG secondary horseradish peroxidase (HRP)-linked antibody (1:5000; Cell Signaling Technology, Boston, MA, USA), and anti-mouse IgG secondary HRP-linked antibody (1:5000; Thermo Fisher Scientific).

### 2.8. Fluorescent Albumin Purification Assay

Conditioned MDA-MB-231 cell culture media (4 mL) was spiked with bovine serum albumin (BSA) alexa fluor 488 conjugate (60 μg; Thermo Fisher Scientific). The media was then processed using UC or TFF, and the fluorescence intensity of the samples (100 μL) was measured (Ex 485/Em 528) using a plate reader (Synergy HT; Biotek, Winooski, VT, USA). Albumin content was determined based on a fluorescent standard curve. Experiments were performed in triplicate.

### 2.9. Mycoplasma Assay

Potential mycoplasma contamination was determined using a MycoAlert mycoplasma detection kit (Lonza, Allendale, NJ, USA), according to the manufacturer’s instructions, and luminescence was measured with a plate reader (Synergy HT; Biotek). Mycoplasma detection was performed on three cell culture media-derived EV samples that were isolated from separate TFF runs.

### 2.10. Sterility Assay

The sterility of EV samples that were isolated from cell culture media was evaluated. Samples (100 µL) were inoculated in liquid media (BACTEC bottles; BD) and monitored for aerobic or anaerobic bacterial growth (14 days). Sterility testing was performed on three cell culture media-derived EV samples isolated from separate TFF runs.

### 2.11. Endotoxin Assay

Endotoxin quantification was performed using a Toxin Sensor Chromogenic LAL Endotoxin Assay Kit (Gen Script, Piscataway, NJ, USA), according to the manufacturer’s instructions, and the absorbance was measured using a plate reader (Synergy HT; Biotek). Endotoxin detection was performed on three cell culture media-derived EV samples that were isolated from separate TFF runs.

### 2.12. EV Track

All relevant data has been submitted to the EV-TRACK knowledgebase (EV-TRACK ID: EV-TRACK code CY5310BC) [34].

## 3. Results

UC is the most common protocol for concentrating EVs from biological fluids [11]. Despite the widespread use of this method, several reports have highlighted the major limitations of this technique, especially in regard to clinical translation of EVs. In the past years, UC protocols have been partially replaced by new techniques, such as FFF and high-performance liquid chromatography (HPLC) that improve EV isolation in regard to time, yield, purity, or scalability.

The concentration efficiency of UC and TFF processing of supernatant preparations from MDA-MB-231 cells was compared. Cell culture media is a commonly used biological fluid that is characterized by a predictable composition. The results, which refer to three independent replicate experiments, are summarized in Figure 1. When compared to UC, TFF resulted in a one to two orders of magnitude increase in EV recovery per million cultured cells (approximately 1010 EVs/106 cells for TFF and 108 EVs/106 cells for UC) (Figure 1b). Accordingly, the yield of EVs from the same amount of media (100 mL) was improved approximately five-fold with TFF (Figure 1b).

Essential biophysical and biochemical properties (according to the standards suggested by the International Society of Extracellular Vesicles [15]) of the EVs that were processed with the two methods were determined and compared. The size distribution of EVs that were obtained by UC and TFF ranged from 60–600 nm (Figure 1c and Supplementary Figure S1a), with a comparable mean between 140–210 nm (Supplementary Figure S2a). TFF give rise to less discrete distribution peaks of EVs as compared to UC. However, the TFF size distribution profiles are more similar among the three replicates, suggesting higher batch-to-batch reproducibility, which is a highly desirable feature. Transmission electron microscopy (TEM) also revealed that both methods resulted in samples that are enriched in nanosized particles with a diameter ranging from 50–200 nm and with spherical morphology, characteristic of EVs (Figure 1d,e). It is worth noting that less EVs were seen in UC samples (Figure 1d) as compared to TFF samples (Figure 1e)

Samples were also analyzed with Western blot to identify characteristic EV markers. The results indicate that CD63 and CD81 were present in the samples and enriched 25.2 and 15.3-fold when compared to cell homogenate (Figure 1f and Supplementary Figure S3a). Calnexin, an endoplasmic reticulum marker that is used as a control to detect intracellular vesicle contaminants, was undetectable (Figure 1f and Supplementary Figure S3a).

Furthermore, the ability of both methods to separate contaminants with typical protein size was assayed by spiking the samples with known amounts of albumin (14.1 × 4.2 nm [35]). The results reveal that TFF leads to a 40-fold improvement in the ability to remove albumin when compared to UC (25% uncertainty) (Figure 1g). Moreover, the albumin concentration differed substantially among the three samples that were processed by UC (Figure 1g), highlighting a lack of batch-to-batch consistency.

In regard to clinical translation, it is important to determine whether sterility can be maintained throughout the isolation process. Therefore, the EV-enriched samples that were isolated with TFF underwent mycoplasma, bacteria, and endotoxin testing. All of the samples were negative for mycoplasma and bacteria, and they displayed acceptable levels of endotoxin (Table 1).

In conclusion, TFF resulted in higher EV yield, improved batch-to-batch consistency, and less albumin contaminants when compared to UC. Moreover, TFF is a time-efficient (~1 h for 200 mL cell culture media), scalable, and sterile method for obtaining EV-enriched samples.

Next, the ability of TFF to concentrate EVs from patient-derived biological fluids (lipoaspirate-derived fluids) was evaluated. EVs derived from adipose tissue have clinical relevance as therapeutics, diagnostics, and cosmetics. Lipoaspirate is a heterogeneous mixture of cells, cell fragments, and other micron-/nano-sized components obtained through liposuction. Lipoaspirate processing with the United States Food and Drug Administration (FDA) approved the Lipogems device (see Section 2), separates cells from other components, which are collected in a sterile waste bag [28]. The acellular fluid in the waste bag consists of a heterogeneous mixture of nano-sized particles and micron-sized soft lipid particles that resemble milk fat globules. The UC of this fluid would lead to fragmentation of the fragile microparticles into nanoparticles that would be co-concentrated with the EVs [36,37]. On the contrary, TFF is operated under controlled low pressure and flow conditions [38], providing a suitable method to gently sieve out microparticles, avoiding sample manipulation that could lead to the formation of artificial nanoparticles during the concentration step.

After processing 50 mL of lipoaspirate through the Lipogems device, the amount of fluids collected into the sterile waste bag was approximately 3 L. Processing this fluid with TFF led to a total EV yield of approximately 10^12^ EVs in 9 mL of buffer. This amount of EVs is suitable for performing extensive functional in vitro assays and therapeutic in vivo studies, as indicated in Table 2.

**Figure 2:**
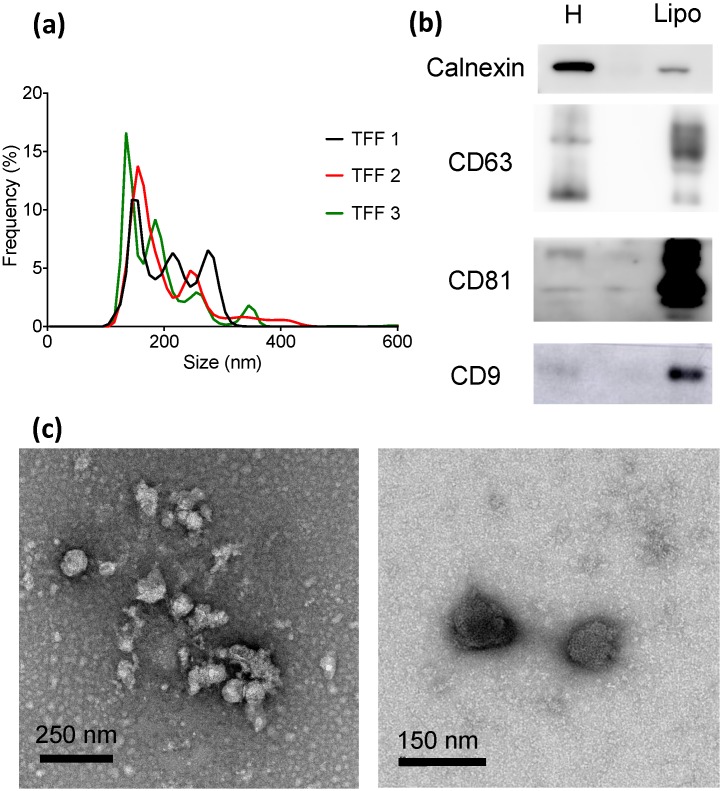
Biophysical and biochemical characterization of EV-enriched lipoaspirate-derived samples (Lipo) isolated with TFF; (**a**) Size distribution of EVs (triplicate); (**b**) Western blot of characteristic intracellular (calnexin) and EV (CD63, CD81, CD9) markers. Cellular homogenate (H) was used as a control; (**c**) TEM images of EV-enriched formulations.

The size distribution of the samples ranged from 70 to 650 nm (Figure 2a and Supplementary Figure S1b), with a mean size between 170–185 nm (Supplementary Figure S2b). Western blot analysis confirmed the presence of EV-enriched markers, such as CD63, CD81, and CD9 (Figure 2b). The presence of calnexin, an endoplasmic reticulum protein that is used as a marker of intracellular membrane fragments and/or membranous micro- and nanoparticles that are released upon cell lysis, was also assessed by Western blot. When compared to cell homogenates, lipoaspirate-derived EV-enriched samples had a much lower calnexin amount (Figure 2b). Indeed, the intensity of the calnexin band was five-fold higher in cell homogenates as compared to lipoaspirate samples, while the signal intensity of EV-enriched marker bands was 1.5 (CD63), 6.9 (CD81), and 2.7-fold (CD9) higher in lipoaspirate samples (Supplementary Figure S3b). These results indicate that the vast majority of nanostructures in the lipoaspirate-derived samples are not formed by cell lysis.

## 4. Conclusions

This is a proof of concept study that indicates that TFF is an efficient method for obtaining EV-enriched formulations from large volumes of biological fluids that are characterized by a rarefied EV content in a robust, scalable, time-efficient, and reproducible manner. In particular, TFF was able to concentrate EVs from fluids derived from patient lipoaspirate, which can represent a valuable cell-free option with regenerative potential.

Side-by-side comparison demonstrated that TFF outperformed UC in all parameters measured in this study, including yield, removal of single macromolecules and aggregates (<15 nm), and batch-to-batch consistency. A comparison of the functional activity of EVs concentrated by the two techniques will be fundamental for the further assessment of TFF. In addition, it is worth noting that in the preclinical laboratory environment, EV concentration by TFF is likely to involve higher costs than UC, in terms of hardware equipment and the use of disposable filters. Further studies are needed to explore the suitability and performance of TFF for processing other types of patient-derived biological specimens of biomedical interest, such as liquid (e.g., blood) or solid (e.g., tumor) biopsies. However, solid biopsies would require enzymatic digestion [45], which could lead to technical difficulties and/or artifacts due to the addition of a pre-analytical enzymatic digestion step.

## Figures and Tables

**Table 1:** Sterility, mycoplasma, and endotoxin test results.

	Sterility Test	Mycoplasma Test (Negative < 0.9)	Endotoxin Test (EU/mL)
EV sample 1	Negative	0.3	0.1
EV sample 2	Negative	0.4	0.1
EV sample 3	Negative	0.2	0.7

**Table 2:** Preclinical use of extracellular vesicle (EV)-enriched lipoaspirate samples isolated by tangential flow filtration (TFF).

Study Type	EV Dose	EV Amount Isolated from 50 mL of Lipoaspirate	Extent of Studies that Can Be Performed
functional in vitro assays	~10^10^ EVs/10^6^ cells [39,40,41,42]	10^12^	2000 wells in a 96-well plate (max 50,000 cells/well)
therapeutic in vivo studies	~2 × 10^10^ EVs/ mouse (intravenous or intraperitoneal administration) [41,43,44]	10^12^	50 mice with a single administration

## Supplementary Materials

Supplementary materials are available online.

## References

[1] Colombo M., Raposo G., Thery C. (2014). Biogenesis, secretion, and intercellular interactions of exosomes and other extracellular vesicles. Annu. Rev. Cell Dev. Biol..

[2] Candelario K.M., Steindler D.A. (2014). The role of extracellular vesicles in the progression of neurodegenerative disease and cancer. Trends Mol. Med..

[3] Vader P., Breakefield X.O., Wood M.J. (2014). Extracellular vesicles: Emerging targets for cancer therapy. Trends Mol. Med..

[4] Borrelli D.A., Yankson K., Shukla N., Vilanilam G., Ticer T., Wolfram J. (2018). Extracellular vesicle therapeutics for liver disease. J. Control. Release.

[5] Wu Y., Deng W., Klinke D.J. (2015). Exosomes: Improved methods to characterize their morphology, RNA content, and surface protein biomarkers. Analyst.

[6] Paolini L., Zendrini A., Radeghieri A. (2018). Biophysical properties of extracellular vesicles in diagnostics. Biomark. Med..

[7] Conlan R.S., Pisano S., Oliveira M.I., Ferrari M., Mendes Pinto I. (2017). Exosomes as Reconfigurable Therapeutic Systems. Trends Mol. Med..

[8] Gilligan K.E., Dwyer R.M. (2017). Engineering Exosomes for Cancer Therapy. Int. J. Mol. Sci..

[9] Jiang L., Vader P., Schiffelers R.M. (2017). Extracellular vesicles for nucleic acid delivery: Progress and prospects for safe RNA-based gene therapy. Gene Ther..

[10] Karimi N., Cvjetkovic A., Jang S.C., Crescitelli R., Hosseinpour Feizi M.A., Nieuwland R., Lotvall J., Lasser C. (2018). Detailed analysis of the plasma extracellular vesicle proteome after separation from lipoproteins. Cell. Mol. Life Sci..

[11] Gardiner C., Di Vizio D., Sahoo S., Thery C., Witwer K.W., Wauben M., Hill A.F. (2016). Techniques used for the isolation and characterization of extracellular vesicles: Results of a worldwide survey. J. Extracell. Vesicles.

[12] Furi I., Momen-Heravi F., Szabo G. (2017). Extracellular vesicle isolation: Present and future. Ann. Transl. Med..

[13] Busatto S., Giacomini A., Montis C., Ronca R., Bergese P. (2018). Uptake Profiles of Human Serum Exosomes by Murine and Human Tumor Cells through Combined Use of Colloidal Nanoplasmonics and Flow Cytofluorimetric Analysis. Anal. Chem..

[14] Gamez-Valero A., Monguio-Tortajada M., Carreras-Planella L., Franquesa M., Beyer K., Borras F.E. (2016). Size-Exclusion Chromatography-based isolation minimally alters Extracellular Vesicles’ characteristics compared to precipitating agents. Sci. Rep..

[15] Théry C., Witwer K.W., Aikawa E., Alcaraz M.J., Anderson J.D., Andriantsitohaina R., Antoniou A., Arab T., Archer F., Atkin-Smith G.K. (2018). Minimal information for studies of extracellular vesicles 2018 (MISEV2018): A position statement of the International Society for Extracellular Vesicles and update of the MISEV2014 guidelines. J. Extracell. Vesicles.

[16] Baranyai T., Herczeg K., Onodi Z., Voszka I., Modos K., Marton N., Nagy G., Mager I., Wood M.J., El Andaloussi S. (2015). Isolation of Exosomes from Blood Plasma: Qualitative and Quantitative Comparison of Ultracentrifugation and Size Exclusion Chromatography Methods. PLoS ONE.

[17] Witwer K.W., Buzas E.I., Bemis L.T., Bora A., Lasser C., Lotvall J., Nolte-’t Hoen E.N., Piper M.G., Sivaraman S., Skog J. (2013). Standardization of sample collection, isolation and analysis methods in extracellular vesicle research. J. Extracell. Vesicles.

[18] Coumans F.A.W., Brisson A.R., Buzas E.I., Dignat-George F., Drees E.E.E., El-Andaloussi S., Emanueli C., Gasecka A., Hendrix A., Hill A.F. (2017). Methodological Guidelines to Study Extracellular Vesicles. Circ. Res..

[19] Ismail N., Wang Y., Dakhlallah D., Moldovan L., Agarwal K., Batte K., Shah P., Wisler J., Eubank T.D., Tridandapani S. (2013). Macrophage microvesicles induce macrophage differentiation and miR-223 transfer. Blood.

[20] Paolini L., Zendrini A., Di Noto G., Busatto S., Lottini E., Radeghieri A., Dossi A., Caneschi A., Ricotta D., Bergese P. (2016). Residual matrix from different separation techniques impacts exosome biological activity. Sci. Rep..

[21] Giddings J.C., Yang F.J., Myers M.N. (1976). Flow-field-flow fractionation: A versatile new separation method. Science.

[22] Yang J.S., Lee J.C., Byeon S.K., Rha K.H., Moon M.H. (2017). Size Dependent Lipidomic Analysis of Urinary Exosomes from Patients with Prostate Cancer by Flow Field-Flow Fractionation and Nanoflow Liquid Chromatography-Tandem Mass Spectrometry. Anal. Chem..

[23] Zhang H., Freitas D., Kim H.S., Fabijanic K., Li Z., Chen H., Mark M.T., Molina H., Martin A.B., Bojmar L. (2018). Identification of distinct nanoparticles and subsets of extracellular vesicles by asymmetric flow field-flow fractionation. Nat. Cell Biol..

[24] Sitar S., Kejzar A., Pahovnik D., Kogej K., Tusek-Znidaric M., Lenassi M., Zagar E. (2015). Size characterization and quantification of exosomes by asymmetrical-flow field-flow fractionation. Anal. Chem..

[25] Frese L., Dijkman P.E., Hoerstrup S.P. (2016). Adipose Tissue-Derived Stem Cells in Regenerative Medicine. Trans. Med. Hemother..

[26] Rani S., Ryan A.E., Griffin M.D., Ritter T. (2015). Mesenchymal Stem Cell-derived Extracellular Vesicles: Toward Cell-free Therapeutic Applications. Mol. Ther..

[27] Kogure T., Lin W.L., Yan I.K., Braconi C., Patel T. (2011). Intercellular nanovesicle-mediated microRNA transfer: A mechanism of environmental modulation of hepatocellular cancer cell growth. Hepatology.

[28] Tremolada C., Colombo V., Ventura C. (2016). Adipose Tissue and Mesenchymal Stem Cells: State of the Art and Lipogems(R) Technology Development. Curr. Stem Cell Rep..

[29] Ungerechts G., Bossow S., Leuchs B., Holm P.S., Rommelaere J., Coffey M., Coffin R., Bell J., Nettelbeck D.M. (2016). Moving oncolytic viruses into the clinic: Clinical-grade production, purification, and characterization of diverse oncolytic viruses. Mol. Ther. Methods Clin. Dev..

[30] Blechacz B., Splinter P.L., Greiner S., Myers R., Peng K.W., Federspiel M.J., Russell S.J., LaRusso N.F. (2006). Engineered measles virus as a novel oncolytic viral therapy system for hepatocellular carcinoma. Hepatology.

[31] Zhang L., Steele M.B., Jenks N., Grell J., Behrens M., Nace R., Naik S., Federspiel M.J., Russell S.J., Peng K.W. (2016). Robust oncolytic virotherapy induces tumor lysis syndrome and associated toxicities in the MPC-11 plasmacytoma model. Mol. Ther..

[32] Langfield K.K., Walker H.J., Gregory L.C., Federspiel M.J. (2011). Manufacture of measles viruses. Methods Mol. Biol..

[33] Sviben D., Forcic D., Halassy B., Allmaier G., Marchetti-Deschmann M., Brgles M. (2018). Mass spectrometry-based investigation of measles and mumps virus proteome. Virol. J..

[34] Consortium E.-T., Van Deun J., Mestdagh P., Agostinis P., Akay O., Anand S., Anckaert J., Martinez Z.A., Baetens T., Beghein E. (2017). EV-TRACK: Transparent reporting and centralizing knowledge in extracellular vesicle research. Nat. Methods.

[35] Wright A.K., Thompson M.R. (1975). Hydrodynamic structure of bovine serum albumin determined by transient electric birefringence. Biophys. J..

[36] Zheng H., Jimenez-Flores R., Everett D.W. (2013). Bovine milk fat globule membrane proteins are affected by centrifugal washing processes. J. Agric. Food Chem..

[37] Huppertz T., Kelly A.L. (2006). Physical Chemistry of Milk Fat Globules. Advanced Dairy Chemistry Volume 2 Lipids.

[38] Shankaran H., Neelamegham S. (2001). Effect of secondary flow on biological experiments in the cone-plate viscometer: Methods for estimating collision frequency, wall shear stress and inter-particle interactions in non-linear flow. Biorheology.

[39] Gai1 C., Gomez Y., Tetta C., Felice Brizzi M., Camussi G. (2017). Protective Role of Stem Cell Derived Extracellular Vesicles in an In Vitro Model of Hyperglycemia-Induced Endothelial Injury. J. Cell Sci. Ther..

[40] Lopatina T., Bruno S., Tetta C., Kalinina N., Porta M., Camussi G. (2014). Platelet-derived growth factor regulates the secretion of extracellular vesicles by adipose mesenchymal stem cells and enhances their angiogenic potential. Cell Commun. Signal..

[41] Cavallari C., Ranghino A., Tapparo M., Cedrino M., Figliolini F., Grange C., Giannachi V., Garneri P., Deregibus M.C., Collino F. (2017). Serum-derived extracellular vesicles (EVs) impact on vascular remodeling and prevent muscle damage in acute hind limb ischemia. Sci. Rep..

[42] De Godoy M.A., Saraiva L.M., de Carvalho L.R.P., Vasconcelos-dos-Santos A., Beiral H.J.V., Ramos A.B., de Paula Silva L.R., Leal R.B., Monteiro V.H.S., Braga C.V. (2018). Mesenchymal stem cells and cell-derived extracellular vesicles protect hippocampal neurons from oxidative stress. J. Biol. Chem..

[43] Hiroaki H., Yan I., Borelli D., Matsuda A., Parasramka M., Shukla N., Lee D., Patel T. (2017). Extracellular vesicle from bone marrow derived mesenchymal stem cells protect against murine hepatic ischemia-reperfusion injury. Liver Transpl..

[44] Haga H., Yan I.K., Takahashi K., Matsuda A., Patel T. (2017). Extracellular vesicles from bone marrow-derived mesenchymal stem cells improve survival from lethal hepatic failure in mice. Stem Cells Transl. Med..

[45] Vella L.J., Scicluna B.J., Cheng L., Bawden E.G., Masters C.L., Ang C.S., Willamson N., McLean C., Barnham K.J., Hill A.F. (2017). A rigorous method to enrich for exosomes from brain tissue. J. Extracell. Vesicles.

