# Supplementary material for: Tangential Flow Filtration for Highly Efficient Concentration of Extracellular Vesicles from Large Volumes of Fluid
Source: Cells. 2018 Dec 16;7(12):273. doi: 10.3390/cells7120273 (PMC6315734; doi:10.3390/cells7120273)
Supplement: Supplementary file 1 [file cells-07-00273-s001.pdf]

Supplementary Material

Tangential flow filtration for highly efficient concentration of extracellular vesicles from large volumes of fluid

Sara Busatto[1,2,*,†], George Vilanilam[1,†], Taylor Ticer[1], Wen-Lang Lin[3], Dennis W. Dickson[3], Shane Shapiro[4], Paolo Bergese[2,5,*] and Joy Wolfram[1,6,*]

[1]*Department of Transplantation Medicine; Department of Physiology and Biomedical Engineering, Mayo Clinic, Jacksonville, Florida 32224, USA.*

[2]*Department of Molecular and Translational Medicine, University of Brescia, 50019 Brescia, Italy.*

[3]*Department of Neuroscience, Mayo Clinic, Jacksonville, Florida 32224, USA.*

[4]*Department of Orthopedic Surgery, Mayo Clinic, Jacksonville, FL 32224, USA.*

[5]*CSGI, Research Center for Colloids and Nanoscience, 50019 Florence, Italy*

[6]*Department of Nanomedicine, Houston Methodist Research Institute, Houston, TX 77030, USA.*

[**]Correspondence: busatto.sara@mayo.edu (SB), paolo.bergese@unibs.it (PB), wolfram.joy@mayo.edu (JW)

[†]These authors contributed equally to this work

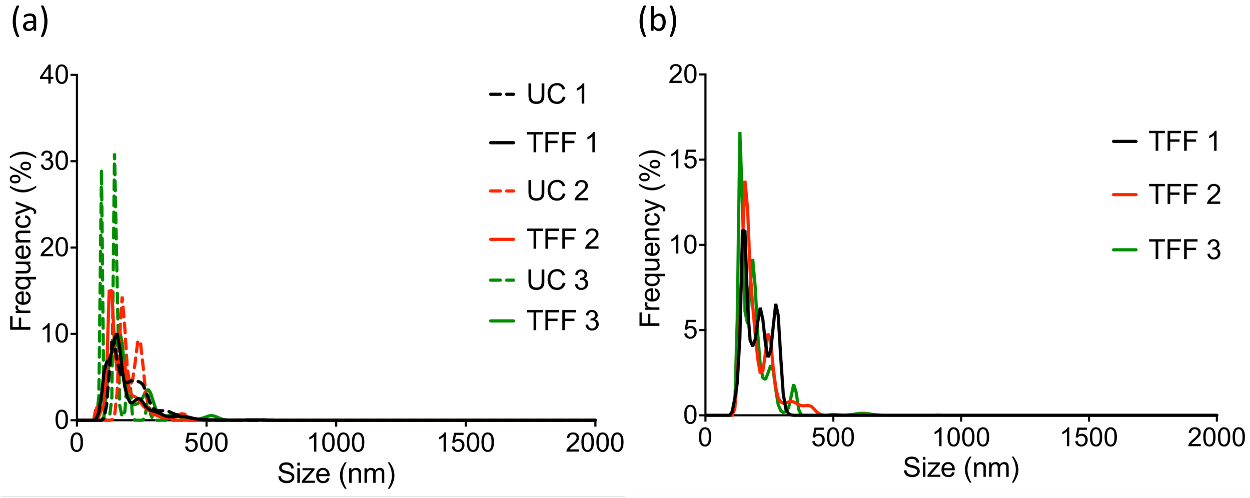

Figure S1. **(a)** Size distribution (0-2000 nm) of extracellular vesicles (EVs) isolated from MDA-MB-231 cell culture media by ultracentrifugation (UC; dashed lines) and tangential flow filtration (TFF; continuous lines). **(b)** Size distribution (0-2000 nm) of EVs isolated from lipoaspirate derived fluids by TFF. Data presented as three technical replicates. Data from figure 1c and figure 2a are redisplayed to illustrate the entire size range that was measured

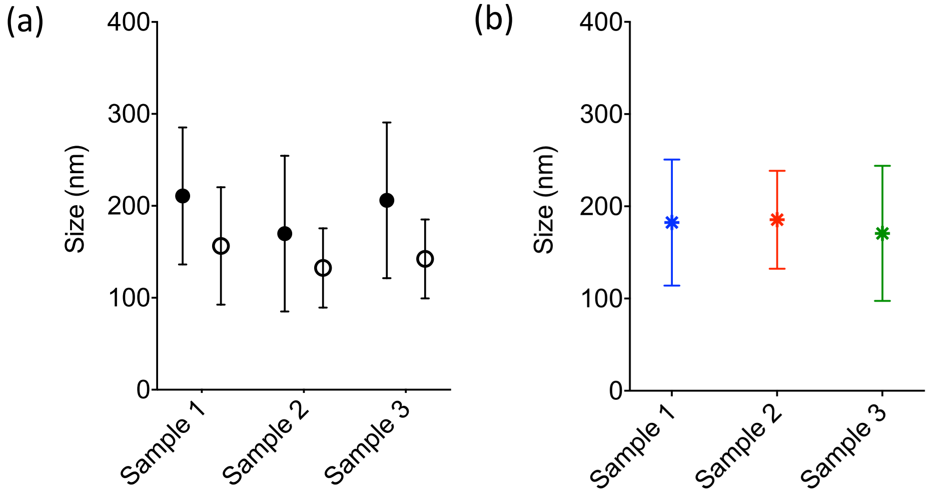

Figure S2. **(a)** Size of EVs isolated from MDA-MB-231 cell culture media by UC (filled circles) and TFF (empty circles). **(b)** Size of EV-enriched fraction concentrated from lipoaspirate derived fluids by TFF. Data are presented as mean ± s.d. of three technical replicates.

(a)

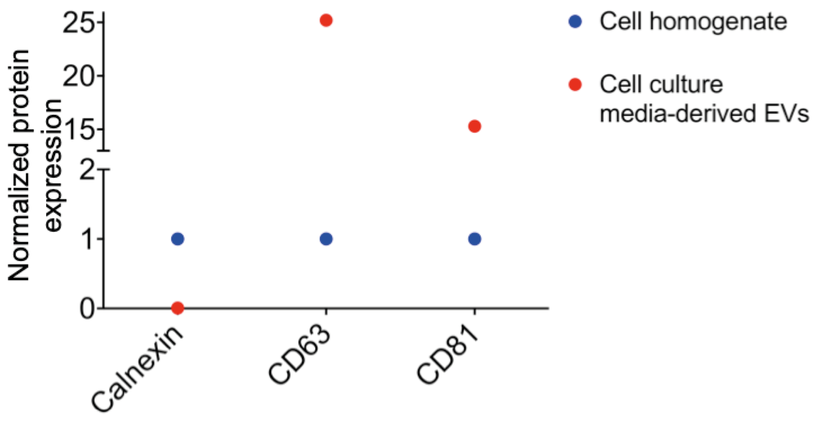

(b)

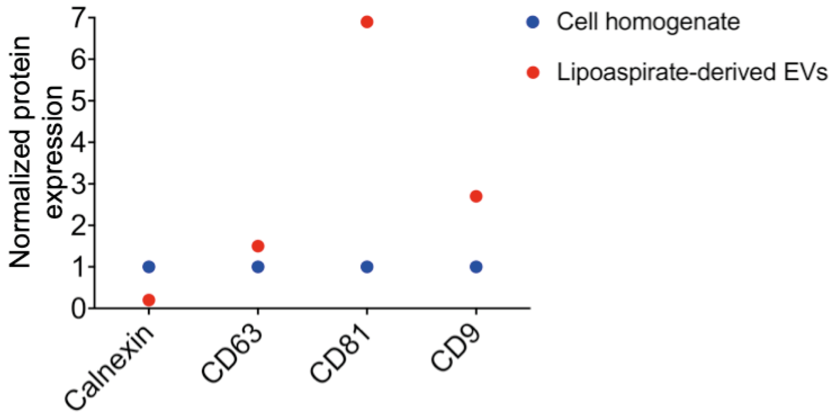

Figure S3. Densitometry analysis of calnexin, CD63, CD81, and CD9 Western blot bands of EV-enriched samples isolated from MDA-MB-231 cell culture media **(a)** or lipoaspirate **(b)** by TFF. Densitometry values were normalized to cell homogenate bands.
